# Functional Neuroanatomy of the Noradrenergic Locus Coeruleus: Its Roles in the Regulation of Arousal and Autonomic Function Part I: Principles of Functional Organisation

**DOI:** 10.2174/157015908785777229

**Published:** 2008-09

**Authors:** E. R Samuels, E Szabadi

**Affiliations:** Psychopharmacology Section, University of Nottingham, Division of Psychiatry, Queen’s Medical Centre, Nottingham, NG7 2UH, UK

**Keywords:** Locus coeruleus, arousal, autonomic function, forebrain, diencephalons, brainstem, spinal cord, autoreceptors.

## Abstract

The locus coeruleus (LC) is the major noradrenergic nucleus of the brain, giving rise to fibres innervating extensive areas throughout the neuraxis. Recent advances in neuroscience have resulted in the unravelling of the neuronal circuits controlling a number of physiological functions in which the LC plays a central role. Two such functions are the regulation of arousal and autonomic activity, which are inseparably linked largely **via** the involvement of the LC. The LC is a major wakefulness-promoting nucleus, resulting from dense excitatory projections to the majority of the cerebral cortex, cholinergic neurones of the basal forebrain, cortically-projecting neurones of the thalamus, serotoninergic neurones of the dorsal raphe and cholinergic neurones of the pedunculopontine and laterodorsal tegmental nucleus, and substantial inhibitory projections to sleep-promoting GABAergic neurones of the basal forebrain and ventrolateral preoptic area. Activation of the LC thus results in the enhancement of alertness through the innervation of these varied nuclei. The importance of the LC in controlling autonomic function results from both direct projections to the spinal cord and projections to autonomic nuclei including the dorsal motor nucleus of the vagus, the nucleus ambiguus, the rostroventrolateral medulla, the Edinger-Westphal nucleus, the caudal raphe, the salivatory nuclei, the paraventricular nucleus, and the amygdala. LC activation produces an increase in sympathetic activity and a decrease in parasympathetic activity **via** these projections. Alterations in LC activity therefore result in complex patterns of neuronal activity throughout the brain, observed as changes in measures of arousal and autonomic function.

## 1 INTRODUCTION

The LC, a pontine nucleus located near the pontomesencephalic junction, is the largest group of noradrenergic neurones in the central nervous system [70, 174, 261]. The LC extensively projects to widespread areas of the brain and spinal cord and it was believed for many years that the outputs of this nucleus formed a diffuse and non-selective contribution to the generalised neural activation underlying themaintenance of arousal [115, 248, 268, 365, 370]. More recently, as the pathways involving the LC have been delineated, it has become clear that the projections of the LC are extremely selective [32, 105, 208, 209, 210]. In addition, the inputs to the LC are extensively varied, contributing to the complex role of this nucleus in a variety of inter-related and distinct functions. 

In this review we aim to give an overview of the connections of the LC relative to two of the functions that these connections sub-serve: arousal and autonomic regulation. A number of good reviews have focused on the neuroanatomy of the LC [8, 32, 105, 243, 261, 379], but none to date have concentrated specifically on the functional pathways involving the LC in the regulation of arousal and autonomic function. It should be noted that although the LC is the preeminent noradrenergic nucleus involved in the regulation of arousal, there are some other noradrenergic nuclei, for example the A1/A5 nuclei, which also contribute to autonomic regulation. Several excellent reviews discuss these other noradrenergic nuclei in detail (for example, see 46, 65, 126, 261). In a companion review we discuss the ways in which unravelling the functional anatomy of the LC system helps with the interpretation of experimental findings involving physiological and pharmacological variables likely to act **via** this system (see Part II).

## 2 OUTPUT FUNCTIONS OF THE LOCUS COERULEUS

Noradrenergic receptors on follower cells receiving an afferent input from the LC can be generally classified as α_1_-, α_2_- or β-adrenoceptors. Activation of α_1_-adrenoceptors by noradrenaline generally leads to excitation of the follower cells [159] and there is some evidence that β-adrenoceptors are also excitatory [32]. In contrast, activation of α_2_-adrenoceptors leads to inhibition of the follower cells [159], and also of the noradrenergic neurones themselves (“autoreceptors”, see 3.2). The consequences of autoreceptors activation can be detected as changes in the firing rate of LC neurones and in the release of noradrenaline. α_2 – _Adrenoceptors are widely distributed in the brain [43,144], and there are regional differences in their role in modulating noradrenaline release [360].

### 2.1 Forebrain

#### 2.1.1 Neocortex (Coeruleo-Cortical Pathway)

The LC extensively innervates the cerebral cortex of all hemispheric lobes [115, 161, 164, 298, 321, 370] and is the sole source of cortical noradrenaline [32, 261]. Indeed, as would be expected, there is a close correlation between LC neurone activity and noradrenaline release within the neocortex [24]. Despite the extensive nature of the projection, there is a substantial specificity within the distribution of LC fibres [32, 198, 246, 247]. It is likely that this noradrenergic input is excitatory, since α_1_-adrenoceptors are expressed in high concentrations throughout the cortex [75, 81, 272, 273, 281]. Interestingly, α_2_-adrenoceptors have also been detected in the neocortex [275, 300, 356, 358], although these tend to be fewer in number than the α_1_-adrenoceptors and distributed more selectively [275, 300, 356]. Surprisingly, activation of these receptors has been found to increase the activity of the neocortex [9]. It has been suggested that these α_2_-adrenoceptors may be present on inhibitory interneurones in the neocortex, where noradrenergic stimulation arising from the LC disinhibits the cortical neurones from inhibition by these interneurones and thus leads to an increase in cortical activity [9]. 

The noradrenergic projection to the neocortex is likely to contribute to the generally recognised role of the LC as a major wakefulness-promoting nucleus (for example, see 256, 257). A number of pieces of converging evidence support this role. Firstly, the activity of the LC closely correlates with level of arousal [14, 15, 16, 103, 104, 287, 291]. Secondly, increases in LC activity have been found to increase EEG signs of cortical arousal [25] whilst LC inactivation results in a reduction in cortical EEG activity [30, 73]. Indeed, increasing the noradrenergic activation of α_1_-adrenoceptors in the prefrontal cortex in rats has been found to increase cognitive performance, and this was interpreted as resulting from an increase in arousal [191]. Finally, electrical stimulation of the LC in a human subject resulted in a reduction in the quantity of both slow wave and rapid eye movement sleep [165], again supporting a wakefulness-promoting role for LC activation.

#### 2.1.2 Basal Forebrain

The BF, comprising the medial septal area, medial preoptic area and substantia innominata, contains both cholinergic and GABAergic cortically-projecting neurones involved in modulating the sleep-wakefulness state [122, 159, 242]. All three areas of the BF receive noradrenergic inputs from the LC [96, 163, 406]. The cholinergic BF neurones are most active during wakefulness, corresponding to an increase in cortical activation, whilst the GABAergic BF neurones are most active during slow-wave sleep and show reduced activity when cortical activation is high [159, 160, 221, 222]. The cholinergic wakefulness-promoting neurones of the BF are activated by noradrenaline released from the terminals of neurones projecting from the LC [96, 106, 159, 160] and this activation is likely to result from stimulation of α_1_- and β-adrenoceptors identified in the medial preoptic and septal areas [26, 27, 31, 106]. Indeed, pharmacological activation of the of α_1_- and β-adrenoceptors in the medial preoptic and septal areas results in an additive increase in arousal above the level achieved by the stimulation of either receptor type alone [27]. In contrast, the GABAergic sleep-promoting neurones of the BF, situated in the medial preoptic area, are inhibited by the stimulation of α_2_-adrenoceptors [223, 242, 356]. Thus, the noradrenergic projection from the LC inhibits these sleep-promoting neurones to promote wakefulness, and these neurones become disinhibited following LC quiescence at the onset of sleep [159, 160, 223]. The overall effect of LC innervation to the cholinergic and GABAergic neurones of the BF is therefore the promotion of arousal. In agreement with this, infusions of noradrenaline into the BF in the rat increase signs of wakefulness [28, 29, 50, 159].

#### 2.1.3 Limbic System

The amygdala is principally responsible for fear and anxiety responses to threatening environmental stimuli, including the increase in the activity of the sympathetic nervous system to threat [71, 174, 192]. The LC densely innervates the amygdala [164, 243] and in particular projects to the central and basal nuclei [98, 163, 261, 354]. The amygdala primarily expresses α_1_-adrenoceptors [75, 81, 272, 273, 281], although α_2_-adrenoceptors have also been detected within this area [117, 275, 300, 356]. These α_2_-adrenoceptors may be located on particular subsets of neurones involved in the autonomic response to stressful stimuli (for example, see 117). The overall noradrenergic influence on the amygdala, however, is likely to be largely excitatory.

Activation of the LC by electrical stimulation or drug administration (for example yohimbine) results in observations of increased anxiety [57, 118, 234, 252, 293, 294] [368], likely as a result of the potentiation of this excitatory pathway from the LC to the amygdala. In addition to a role in anxiety, the LC projection to the amygdala may also play a role in forming and retrieving emotional memories [59, 340]. Interestingly, level of arousal, highly correlated to LC activity (see 2.1.1), determines the likelihood of a memory being encoded and subsequently retrieved. Indeed, both α_1_- and β-adrenoceptors in the basolateral amygdala have been implicated in memory storage [100, 101].

The LC also innervates the hippocampus, providing the sole source of noradrenaline to the hippocampal neurones [110, 174, 204, 208, 210, 261, 277, 369]. Both α_1_-adrenoceptors [271, 281], particularly α_1D_-adrenoceptors [75], and α_2_-adrenoceptors [275, 300] have been detected in the hippocampal formation. This limbic structure is centrally involved in the formation of declarative memories and the LC projection to the hippocampus may thus contribute to memory formation. In support of this possible function, lesions of the LC impair olfactory learning in experimental animals [343]. The LC has also been implicated in memory retrieval [80, 311], although this is likely to be mediated through areas outside of the hippocampus, possibly involving the amygdala [174] (see above).

### 2.2 Diencephalon

#### 2.2.1 Thalamus

The LC sends a large output to the thalamus, especially to the dorsally located nuclei [147, 164, 261]. The principle adrenoceptor identified in the thalamus appears to be the excitatory α_1_-adrenoceptor [75, 281, 409]. Some studies have also reported the presence of α_2_-adrenoceptors in the thalamus [356, 358, 409], although others have not [275, 300]. This output to the thalamus may be related to the wakefulness-promoting role of the LC (see 2.1.1), since thalamic neurones project extensively to the cortex [158, 231]. Indeed, noradrenergic input to the thalamic cells promotes a single spike firing mode of activity in the thalamus that has been related to increased cortical activity and responsiveness during waking [231, 233]. In addition to a role in wakefulness, sparse projections from the LC to the ventral posterolateral nucleus of the thalamus may be involved in modulating the sensation of pain [389].

#### 2.2.2 Hypothalamus

##### 2.2.2.1 Ventrolateral Preoptic Area

In addition to the cortical and thalamic projections described above, the LC contributes to the maintenance of arousal **via** an inhibitory output to the GABAergic neurones of the VLPO of the hypothalamus [62, 242, 270], an area associated with the regulation of sleep-wakefulness state (see Figs. (**1**) and (**4**) in Part II). Neurones of the VLPO exhibit a high activity state during SWS and REM sleep, whilst being virtually silent during periods of waking [354]. This is in contrast to the activity of the LC, where neurones are active during wakefulness, quiet during SWS and quiescent during REM sleep [14, 16, 104, 287]. Noradrenaline, released from the LC during wakefulness, inhibits the majority of VLPO neurones through the stimulation of α_2_-adrenoceptors [114, 228, 241, 270]. When active the VLPO inhibits multiple areas involved with promoting wakefulness, primarily the TMN of the hypothalamus, through GABAergic projections [324]. The TMN sends wakefulness-promoting histaminergic projections to the cerebral cortex [181]; inhibition of the VLPO by the LC thus disinhibits this wakefulness-promoting projection from the TMN. Through this projection to the VLPO and the excitatory projections to the cortex and thalamus (see 2.1.1 and 2.2.1) the LC plays an important role in maintaining arousal [256, 257].

##### 2.2.2.2 Paraventricular Nucleus

A second hypothalamic area to receive a significant projection from the LC is the PVN, a major premotor autonomic nucleus associated with both the sympathetic and parasympathetic nervous systems [163, 164, 261, 350, 351]. Autonomic projections from the PVN terminate on sympathetic preganglionic neurones in the IML of the spinal cord [35, 310, 317, 350, 377] and on parasympathetic preganglionic nuclei in the brainstem [35, 44, 137, 317, 391, 409]. Expression of α_1_-adrenoceptors has been detected in the PVN [3, 75, 273, 281, 309, 342] and the activation of these receptors has been linked to the autonomic response to stress [309, 342]. In addition, α_2_-adrenoceptors have been detected on inhibitory GABAergic neurones synapsing with these spinally-projecting PVN neurones, providing a further means of activating the autonomic nervous system through noradrenaline release from the LC [200]. Through this projection, therefore, the LC influences functions of the autonomic nervous system relating to behavioural arousal, for example cardiovascular regulation (suppression of the baroreceptor reflex: 145, 326). 

The LC is also involved in neuroendocrine function by projections to the neuroendocrine cells of the PVN and tuberoinfundibular nucleus of the hypothalamus (see 2.2.2.4). These neuroendocrine cells are responsible for the secretion of trope hormones, for example thyrotropin-releasing hormone and somatostatin, modulating pituitary activity (see 305).

##### 2.2.2.3 Lateral Hypothalamus/Perifornical Area

The orexin (also called hypocretin) neurones of the LH/PF receive a noradrenergic input from the LC [18, 202, 398]. In contrast to the excitatory influence of orexin on the LC (see 3.1.3.3, below), this noradrenergic input inhibits LH/PF firing [202, 398]. This suggests that there is a negative feedback circuit from the LC to the LH/PF, likely to be involved in preventing excessive activity in the arousal pathway during periods of wakefulness. Some excitatory α_1_-adrenoceptors have also been identified in the LH/PF [3, 281, 342], where activation of these receptors has been linked to behavioural activation and exploration [342]. However, many reports on α_1_-adrenoceptor localisation do not detect significant numbers in the LH/PF [75, 81, 272]. Interestingly, β-adrenoceptors in the LH/PF appear to be related to noradrenergic suppression of feeding [51, 184].

##### 2.2.2.4 Tuberoinfundibular Area

The LC projects to the arcuate nucleus of the tuberoinfundibular area, the neurones of which are involved in neuroendocrine regulation [123, 167]. Both excitatory α_1_- and β-adrenoceptors [3, 167] and inhibitory α_2_-adrenoceptors [404] have been detected in the arcuate nucleus. The LC may contribute to the regulation of neuroendocrine function **via** these adrenoceptors along with the projection to the PVN (see 2.2.2.2). For example, the arcuate nucleus neurones controlling the secretion of GHRH, which increases GH release from the pituitary [249], are modulated by α_2_-adrenoceptor stimulation [64, 404]. Similarly, the arcuate nucleus is involved in the regulation of prolactin secretion **via** the release of dopamine onto the lactotropes in the pituitary [23, 295], which inhibits prolactin secretion. Noradrenergic stimulation of α_1_-adrenoceptors activates these dopaminergic arcuate nucleus neurones and thus contributes to the regulation of prolactin secretion [141]. Indeed, administration of modafinil, a wakefulness-promoting drug believed to enhance noradrenergic LC activity [139] and therefore noradrenaline release onto the dopaminergic neurones, was found to reduce prolactin secretion in healthy male volunteers [305].

### 2.3 Brainstem

#### 2.3.1 Parasympathetic Preganglionic Nuclei

In general, the LC inhibits parasympathetic preganglionic nuclei (Edinger-Westphal nucleus, salivatory nuclei, and vagal nuclei) **via** the activation of inhibitory α_2_-adrenoceptors on the neuronal membrane of the preganglionic follower cells (see Figs. (**2**) and (**4**) in Part II).

##### 2.3.1.1 Edinger-Westphal Nucleus (Coeruleo-Pupillomotor Pathway)

The EWN, the parasympathetic preganglionic nucleus responsible for pupil constriction, receives an ascending input from the LC [42, 207], which is likely to exert an inhibitory influence **via** α_2_-adrenoceptors [133, 134, 352](see Fig. (**2**) in Part II). In the pathway controlling pupil constriction, light stimulation is detected at the retina and transmitted **via** the pretectal nucleus to the EWN [205]. The EWN, located in the oculomotor complex of the midbrain, innervates the ciliary ganglion supplying the sphincter pupillae muscle: activation of this muscle results in constriction of the pupil and a reduction in the level of luminance entering the eye. Through this pathway the EWN mediates both the constriction of the pupil in situations of high background luminance and the reflex constriction of the pupil to a sudden increase in luminance level (light reflex response) [175, 181]. 

The functional significance of the projection from the LC to the EWN is highlighted by two different observations. Firstly, the activation of the LC attenuates the light reflex response (see 2.2.2, Part II). Secondly, there is a species difference in the response of the pupil to α_2_-adrenoceptor agonists, likely to result from a differential activation of α_2_-adrenoceptors within the LC and EWN (see 3.1.1.7, Part II).

##### 2.3.1.2 Salivatory Nuclei (Coeruleo-Salivatory pathway)

The salivatory nuclei are located in the reticular formation, with cells situated ventrolaterally designated as the inferior salivatory nucleus and cells situated dorsolaterally designated as the superior salivatory nucleus [259]. The inferior and superior salivatory nuclei are so divided according to their projections to the periphery: the inferior salivatory nucleus projects through the glossopharyngeal nerve whilst the superior salivatory nucleus projects through the facial nerve. The inferior salivatory nucleus is responsible for the parasympathetic innervation of the otic ganglion, from which postganglionic fibres innervate the parotid and lingual (von Ebner) salivatory glands involved in salivation and taste perception respectively [41, 111, 175]. The superior salivatory gland is responsible for parasympathetic innervation of the submandibular ganglion, from which postganglionic fibres innervate the submandibular and sublingual salivatory glands [61, 151, 319, 405] and the pterygopalatine ganglion innervating the lacrimal gland involved in tear secretion [258, 365]. The neurones of the superior salivatory nucleus may be divided into two categories: type I neurones are responsible for salivation whilst type II neurones are involved in vasodilatation [229].

There is limited information regarding the innervation of the salivatory nuclei, but that which is available suggests a possible role for the LC in modulating these nuclei, and thus contributing to the autonomic control of salivation. The neurones of the superior salivatory nuclei receive synaptic inputs from monoaminergic cell groups [258] and within this there may be a noradrenergic contribution originating in the LC [151, 338]. Inhibitory α_2_-adrenoceptors are located on the preganglionic parasympathetic neurones of the salivatory nuclei [214, 353] and it has been suggested that salivation is tonically inhibited **via** the activation of these receptors [355]. Indeed, there is pharmacological evidence consistent with the existence of inhibitory α_2_-adrenoceptors on salivatory neurones (see 3.1.1.4, Part II).

##### 2.3.1.3 Parasympathetic Vagal Nuclei (Coeruleo-Vagal Pathway)

The parasympathetic vagal nuclei include the DMV and the nucleus ambiguus. The DMV, the largest preganglionic parasympathetic nucleus in the brainstem, has efferents contributing to the control of smooth muscle in the thoracic and abdominal viscera [174]. Along with the nucleus ambiguus (see below), the DMV mediates the parasympathetic control of cardiovascular function [264]. The neurones of the DMV fire in synchrony with the cardiac cycle to reduce heart rate and blood pressure: excitatory cyclical input from the baroreceptors, mediated through the nucleus of the solitary tract, increases the firing of neurones in the DMV [72]. Indeed, microinjections of glutamate into the DMV were found to reduce both heart rate and blood pressure [58]. The LC projects to the DMV [261, 359, 388] and α_2_-adrenoceptors have been detected on DMV neurones [275, 299, 300, 370]. It has been shown that noradrenaline inhibits the activity of DMV neurones **via** the activation of these α_2_-adrenoceptors [112, 226]. Furthermore, microinjection of glutamate into the LC was found to increase HR and BP [58], consistent with an inhibitory influence of the LC on the DMV. This observation is also consistent with the combined sympathomimetic and parasympatholytic effect of LC activation on cardiovascular function.

The nucleus ambiguus innervates the cardiac ganglia to contribute to the control of heart rate [174, 218] and is critical for the heart rate response to baroreceptor stimulation [60]. Chemical stimulation of the neurones of the nucleus ambiguus results in a reduction in heart rate [225] and blood pressure [58]. The nucleus ambiguus also reduces heart rate through an inhibitory projection to the rostroventrolateral medulla [236], the major pre-sympathetic nucleus involved in cardiovascular regulation (see 2.3.2.1). The LC projects to the nucleus ambiguus [164, 388] and α_1_-adrenoceptors have been detected within this nucleus [75]. The presence of α_1_-adrenoceptors in this nucleus appears to be paradoxical since α_1_-adrenoceptors usually mediate excitation and the LC generally exerts an inhibitory effect on preganglionic cholinergic neurones. However, the cellular localisation of these α_1_-adrenoceptors has not been identified and it is possible that they are located on inhibitory interneurones rather than on the preganglionic cholinergic output neurones themselves, as would be consistent with the general pattern of the modulation of autonomic activity by the LC.

#### 2.3.2 Premotor Sympathetic Nuclei

##### 2.3.2.1 Rostroventrolateral Medulla (Coeruleo-Vasomotor Pathway)

In addition to the modulation of parasympathetic nuclei (see 2.3.1.3), the LC contributes to the regulation of cardiovascular function **via** inhibitory projections to the RVLM [376] (see Figs. (**2**) and (**4**) in Part II). The RVLM tonically projects to sympathetic preganglionic neurones in the IML of the spinal cord [19, 53, 72, 344, 346, 407], where glutamate release excites the neurones and promotes vasoconstriction. Some of the spinal-projecting RVLM neurones have an intrinsic pacemaker activity that contributes to the maintenance of normal blood pressure and heart rate [72, 125, 344]. The RVLM neurones are also involved in the response of cardiovascular activity to changing environmental demands, mediated **via** the baroreflex. Baroreceptor activation triggered by an increase in blood pressure enhances nucleus of the solitary tract neurone activity, which activates the GABAergic caudal ventrolateral medulla neurones that inhibit RVLM activity [48, 72, 344, 345, 346]. The activation of this pathway mediates a reduction in cardiovascular activity. In this way, RVLM neuronal activity is synchronised to the cardiac-related rhythm in sympathetic activity [19], with reduced firing rate following baroreceptor activation. In contrast, hypotension increases RVLM activity [119]. In general, chemical or electrical stimulation of the RVLM produces an increase in blood pressure and heart rate, while chemical lesions of the RVLM reduce blood pressure [48, 72, 125, 168, 344, 392]. The inhibition of the RVLM by the LC is likely to result from the stimulation of α_2_-adrenoceptors located within the RVLM [132, 173, 319, 358]. For the functional significance of these receptors see section 3.1.1.3, Part II.

The combined influences of the LC on the sympathetic output to the cardiovascular system (coeruleo-vasomotor and coeruleo-spinal pathways) result in a moderate increase in blood pressure and heart rate when the LC is activated [82, 124, 203, 331, 341, 385], indicating the predominance of the direct innervation of the spinal cord (see 2.5.3). The inhibition of the DMV and nucleus ambiguus (coeruleo-vagal pathway) may also contribute to this effect (see 2.3.1.3). Hypertension has been found to increase GABA release in the LC [332], leading to a reduction in LC neurone activity [248] and a decrease in noradrenaline release [332]. In contrast, hypotension has been found to decrease GABA release in the LC [332], leading to an increase in LC neurone activity [10, 362, 372] and an increase in noradrenaline release [332]. It thus appears that the excitation of the spinal cord and possibly the inhibition of the DMV and nucleus ambiguus by the LC predominate over the inhibition of the RVLM.

##### 2.3.2.2 Caudal Raphe Nuclei

The nuclei of the CR (raphe magnus, obscurus, and pallidus) are innervated by noradrenergic projections from the LC [136, 164], which are likely to have primarily excitatory effects **via** α_1_-adrenoceptor activation [81, 271]. However, α_2_-adrenoceptors have also been identified within the CR [127, 300, 370], suggesting that a subset of CR neurones may be inhibited by the LC projection. The CR is involved in modulating sympathetic function **via** serotonergic outputs to the IML of the spinal cord [7, 150, 206]. Both the raphe pallidus and the raphe magnus have been implicated as excitatory premotor neurones in the regulation of body temperature, innervating sympathetic preganglionic neurones in the IML [255]. This is supported by studies finding that suppression of the activity of the raphe pallidus with microinjection of muscimol, a GABA receptor agonist, results in hypothermia [408] and that cold exposure increases neurone activity within the CR, measurable both as an increase in electrical unit activity [292] and as an increase in Fos expression [36, 245, 254]. Therefore, the excitation of these CR neurones by the projection from the LC would be expected to increase body temperature **via** the activation of the sympathetic nervous system.

Another major action of the serotonergic projection to the spinal cord is the suppression of nociception [66, 113, 239], achieved through the release of serotonin in the dorsal spinal cord [135, 162, 188, 297]. Interestingly, α_1_- and α_2_-adrenoceptors have been found to be co-localised on raphe magnus neurones involved in the inhibition of nociception [34]. These receptors have been implicated in the mechanism underlying opioid-induced analgesia, since blocking the α_1_-adrenoceptors or stimulating the α_2_-adrenoceptors attenuated the analgesia produced through local opioid administration [34]. Thus, the innervation by the LC is likely to be acting at excitatory α_1_-adrenoceptors to increase CR neurone activity and thus contribute to the suppression of nociception during opioid analgesia. 

The projection from the LC to the CR is therefore involved in modulating both the premotor sympathetic neurones involved in thermoregulation and the neurones involved in nociception.

#### 2.3.3 Dorsal Raphe Nucleus

In addition to the projection to the neurones of the CR described above (see 2.3.2.2), the LC also innervates the serotonergic neurones of the DR nucleus [176, 197, 224, 261, 304]. The DR is involved in the regulation of the sleep-wakefulness state and DR serotonergic neurones have been found to fire extensively during wakefulness whilst showing quiescence during periods of sleep [235, 366]. The input from the LC to the DR appears to be excitatory **via** activation of α_1_-adrenoceptors [43, 75, 76, 271, 272, 281, 284, 342, 402] and it may be involved in the maintenance of increased DR neurone firing during wakefulness. Indeed, noradrenaline perfusion directly into the DR results in cortical desynchronisation [172].

#### 2.3.4 Pedunculopontine and Laterodorsal Tegmental Nuclei

The cholinergic neurones of the PPT and LDT are associated with the regulation of the sleep-wakefulness state. The neurones of the PPT and LDT are active during both wakefulness and REM sleep [89, 160, 171]. These neurones project to the thalamus, where they excite neurones involved in promoting cortical desynchrony [160, 169]. Two populations of neurones have been proposed within these nuclei: one set of cholinergic neurones are active during waking and excited by noradrenaline from the LC acting at α_1_-adrenoceptors, and one set of cholinergic neurones that are active during REM sleep and inhibited by noradrenaline from the LC acting at α_2_-adrenoceptors [139, 140]. This second population of neurones may be largely responsible for the initiation of REM sleep [303]; during wakefulness REM sleep is inhibited by the activation of α_2_-adrenoceptors on these neurones [20].

#### 2.3.5 Motor Nuclei

The LC projects to motoneurones in the brainstem and the spinal cord, facilitating motoneurone activity **via** the stimulation of α_1_-adrenoceptors.

##### 2.3.5.1 Facial Nucleus

The LC densely projects to the motoneurones of the facial nucleus [164, 231], a group of motoneurones located at the pontomedullary junction, and this projection appears to be excitatory since extracellular microiontophoretic application of noradrenaline increases the activity of these motoneurones [289, 378, 390]. Indeed, excitatory α_1_-adrenoceptors have been detected within the facial nucleus [75, 81], supporting this facilitatory action.

There is evidence from studies using the acoustic startle paradigm that the facilitatory projection from the LC to the facial nucleus may be tonically active. The acoustic startle paradigm involves the presentation of a sudden intense auditory stimulus to produce a startle response involving the rapid involuntary contraction of facial and skeletal musculature. The conventional measure of this response is the EMG recording of the orbicularis oculi muscle, involved in the eye blink response, which is innervated by the motoneurones of the facial nucleus. It has been found that the administration of a sedative drug such as clonidine, known to reduce LC activity, results in a reduction in the amplitude of the acoustic startle response [1, 2, 187, 307], whilst the administration of the α_2_-adrenoceptor antagonist yohimbine [148, 333, 357], known to increase LC activity, enhances the amplitude of the response [244].

##### 2.3.5.2 Hypoglossal Nucleus

There is limited information regarding the afferents of the hypoglossal nucleus, but there is some evidence of a noradrenergic influence on hypoglossal motoneurones. Retrograde and anterograde transport techniques have identified a projection to the hypoglossal nucleus from the subcoeruleus nucleus [6], which may include neurones of the LC. Indeed, the descending projection of the LC passes ventrolaterally to the hypoglossal nucleus [231] and thus it is possible that fibres from this pathway innervate the nucleus. Additionally, there are α_1_-adrenoceptors, but not α_2_-adrenoceptors, on these neurones [274, 343] suggesting an excitatory influence of noradrenaline on the nucleus. Application of noradrenaline to the hypoglossal nucleus results in motoneurone activation and the application of an α_1_-adrenoceptor agonist (phenylephrine) mimicked this effect [274]. In contrast, the application of an α_1_-adrenoceptor antagonist (prazosin) prevented the noradrenaline-induced increase in activity [274].

##### 2.3.5.3 Trigeminal Motor Nucleus

Although the majority of noradrenergic projections to the trigeminal motoneurones arise from the A5/A7 nuclei, the LC also projects to this nucleus, albeit sparsely [217]. In general, noradrenaline has a facilitatory influence on these motoneurones [323]. Bilateral locus coeruleus lesions do not significantly reduce noradrenaline content in the trigeminal motor nucleus, suggesting that this input from the LC is of minor physiological significance [197].

##### 2.3.5.4 Oculomotor Nuclear Complex

The motor neurones situated in the nuclei of the third (oculomotor), fourth (trochlear), and sixth (abducens) cranial nerves form the oculomotor nuclear complex responsible for innervating the external muscles of the eyes controlling the movements of the eye. A small number of cells in the LC have been found to project to the oculomotor nuclear complex [52] and high levels of α_1_-adrenoceptors have been identified within this area [75, 81, 281], indicating an excitatory noradrenergic input to these neurones. 

#### 2.3.6 Sensory Nuclei

##### 2.3.6.1 Trigeminal Sensory Nucleus

In contrast to the sparse innervation of the trigeminal motor nucleus, the LC densely innervates the neurones of the trigeminal sensory nucleus [66, 197, 261, 321] and this pathway is likely to be involved in the antinociceptive function of the LC [47, 66, 367]. Indeed, electrical stimulation of the LC has been found to inhibit the neurones of the sensory trigeminal nucleus involved in pain perception [230, 312, 314, 367]. In addition, inhibitory α_2_-adrenoceptors have been detected within the trigeminal nucleus [410]. It has been proposed that β-adrenoceptors may also be involved in the inhibitory influence of noradrenaline on the trigeminal sensory neurones [313]. Recently it has been shown that there is an intricate synergistic interaction between the antinociceptive effects of the noradrenergic and serotonergic inputs to the sensory trigeminal nucleus [66].

##### 2.3.6.2 Cochlear Nucleus

The LC projects diffusely to the cochlear nuclei and noradrenaline levels are detectable here in moderate concentrations [180, 185, 188, 320, 341]. This input is suggested to be excitatory, where noradrenaline enhances both spontaneous and tone-evoked cochlear nucleus neurone activity, and it is likely that this effect is mediated **via** α_1_-adrenoceptors [84]. The LC may, therefore, be involved in sensory auditory processing.

Noradrenergic modulation of cochlear nucleus activity may underlie the mechanism by which clonidine reduces the amplitude of auditory evoked potentials (N1/P2) recorded in the EEG during the acoustic startle paradigm [307] since clonidine is known to act by reducing LC activity. It should be noted, however, that this effect of clonidine on the auditory evoked potentials has not been observed in every study [2].

### 2.4 Cerebellum (Coeruleo-Cerebellar Pathway)

The cerebellum is responsible for the planning, coordination, and learning of movements, particularly relating to the timing, force and extent of muscle contractions, and may also be involved in cognition and emotion [174]. The LC projects to areas throughout the cerebellum [208, 210, 261, 268, 369] and in particular to the cerebellar cortex [301, 320]. A moderate number of α_1_-adrenoceptors has been observed in the cerebellum [75, 271, 273, 318, 342], indicating an excitatory role for the LC in facilitating one or more of the functions of the cerebellum. Indeed, depletion of noradrenaline from the cerebellum has been found to result in impaired motor performance [386].

### 2.5 Spinal Cord (Coeruleo-Spinal Pathway)

The contribution of the LC to autonomic nervous system control involves a direct output to sympathetic and parasympathetic preganglionic neurones of the IML of the spinal cord in addition to the projections innervating other autonomic nuclei, for example the EWN, and premotor autonomic nuclei, for example the PVN, CR, and RVLM, described above (see 2.3.1.1, 2.2.2.2 and 2.3.2.2; see also Figs. (**2**) and (**4**) in Part II). The LC also contributes to sensory and motor functions through projections to the dorsal and ventral horns of the spinal cord, respectively. The LC projects to all three areas (dorsal and ventral horns and IML) to differing extents [131, 164, 196, 38] and there is some evidence that this distribution may differ between rat strains [283, 334].

#### 2.5.1 Dorsal Horn

Neurones in the dorsal horn of the spinal cord are sensory neurones associated with the detection of pain, temperature, touch and position/movement (proprioception) [174]. The LC most densely innervates the cells in this compartment of the spinal cord [63, 107, 108, 265, 283, 387, 388], signifying that the LC can influence the processing of sensory information. This influence is likely to be inhibitory, achieved **via** the activation of pre-synaptic α_2_-adrenoceptors on the terminals of excitatory peptidergic spinal interneurones within the dorsal horn [267]. In support of this suggestion α_2_-adrenoceptors have been identified within this region [189, 275, 325, 336]. The projection from the LC may be particularly important in the processing of noxious stimuli (nociception). Indeed, it has been suggested that the spinal projection from the LC has analgesic properties [331] and α_2_-adrenoceptor agonists acting in the dorsal horn of the spinal cord are known to produce analgesia [88]. Interestingly, α_2_-adrenoceptor antagonists increase the response of dorsal horn neurones to inflammation induced by formalin injection [121]. Additionally, β-adrenoceptors have been detected in the dorsal horn, and may also be involved with nociception [241]. The importance of noradrenaline in the modulation of nociception has been highlighted by the observation of hyperalgesia in the absence of noradrenaline in mice lacking the gene coding for the noradrenaline-synthesising enzyme dopamine-β-hydroxylase [152, 335].

#### 2.5.2 Ventral Horn

The LC sends projections to the neurones of the ventral horn [164, 265, 387], innervating the skeletal musculature, and this pathway may contribute to muscle contraction and tone. Indeed, excitatory α_1_-adrenoceptors are present on the motoneurones of the ventral horn [75, 81, 337], supporting a facilitatory influence of the LC on muscle tone. Both α_2_-adrenoceptors [336] and β-adrenoceptors [241] have also been detected in this region, although the role of these receptors is not clear at present. The loss of LC activity during attacks of cataplexy in narcolepsy [396] may explain the sudden loss of muscle tone characteristic of these attacks [362].

#### 2.5.3 Intermediolateral Cell Column

Neurones of the IML form separate sympathetic preganglionic nuclei that project to ganglia innervating specific target organs. Through these nuclei the sympathetic nervous system can be selectively activated, as opposed to generalised sympathetic activation [11]. The LC has been found to project to the cells of the IML [164, 265, 387] where noradrenaline excites the majority of sympathetic preganglionic neurones [199], possibly **via** the activation of α_1_-adrenoceptors detected in this region [281]. The densest projections from the LC to the IML, however, end in the sacral spinal segments where parasympathetic inhibitory interneurones are located [164, 315, 388, 403] and are involved in functions such as micturition [315]. The projection from the LC to the IML, results in excitation of these inhibitory interneurones **via** the stimulation of α_1_-adrenoceptors [315, 403], which, in turn, leads to a reduction in parasympathetic outflow. There may also be a direct inhibitory influence on the parasympathetic neurones of the IML, since α_2_-adrenoceptors are present within the lumbosacral parasympathetic segments [336, 370, 397]. 

## 3 MODULATION OF LOCUS COERULEUS ACTIVITY

As has become clear, the projections from the LC to the many widespread areas of the neuraxis are complex and extensive. To increase the complexity surrounding this nucleus, the LC also receives multiple varied inputs, which all influence LC firing to differing extents. In the majority of instances the neurotransmitter involved in these inputs to the LC is known; in some however it remains uncertain. 

### 3.1 Modulation by Heteroreceptors **via** Afferent Inputs

A detailed review of the neuroanatomical techniques used to identify afferent inputs to the LC is beyond the scope of this paper, although some of the methods used are covered in Part II. 

#### 3.1.1 Neocortex

Although in general the parietal, temporal, infralimbic, insular and frontal cortices provide only a limited input to the LC [12, 54, 215], there is a strong reciprocal connection between the LC and the prefrontal cortex [153, 154, 332], a cortical area involved in executive functioning. Indeed, the LC has been found to contribute to the regulation of functions such as cognition [32, 38, 39], memory [32], attention [220], and vigilance [120]. The projection from the prefrontal cortex to the LC may provide tonic activation of the LC [154]. Although the neurotransmitter responsible for this activation is unclear, glutamate may be involved since NMDA and non-NMDA excitatory amino acid receptors are present on LC neurones [170, 380].

#### 3.1.2 Amygdala

The LC receives an input from the central nucleus of the amygdala [54, 55, 261, 332, 384], which thus forms the afferent branch of a reciprocal connection between the LC and the amygdala (see 2.1.3). These reciprocal projections may underlie a role for the LC in processing the emotional valence of stimuli. Complementing the increase in anxiety following LC activation (see 2.1.3), states of anxiety induced by stressful and fear-inducing stimuli, including conditioned fear, are accompanied by increases in LC activity [56, 57, 69, 290, 293] (see 2.2.2, part II) and presumably reflect an increase in the activity of this pathway. Additionally, recent evidence has shown that the administration of anxiogenic drugs of different chemical classes (α2 adrenoceptor antagonist, benzodiazepine inverse agonist, 5HT2C receptor agonist, adenosine receptor antagonist, cholecystokinin analogue) leads to an increase in the expression of c-fos activity in the LC [333], whilst the administration of anxiolytic drugs reduces the activity of neurones in the LC [33]. In addition, the projection from the central nucleus of the amygdala to the LC may also be involved in the observed increase in LC activity in response to stressful stimuli [32]. For example, neurones containing CRF in the central nucleus of the amygdala project to the LC and activate these cells in response to stress [375].

#### 3.1.3 Hypothalamus

##### 3.1.3.1 Ventrolateral Preoptic Area

The GABAergic neurones of the VLPO send an inhibitory projection to the LC [54, 193, 216, 279, 331, 339], which, along with the inhibitory projection from the LC to the VLPO (see 2.2.2.1), forms a reciprocal connection between these two areas (see Figs. (**1**) and (**4**) in Part II). During SWS and REM sleep, there is increased release of GABA from the neurones of the VLPO and thus a reduction in the activity of the LC neurones [116, 263]. Indeed, application of GABA to the LC has been found to inhibit cell firing, whilst administration of a GABA receptor antagonist (bicuculline) increases LC activity [116, 219]. In contrast, during wakefulness the inhibitory projection from the LC to the VLPO reduces VLPO neurone activity (see 2.2.2.1) and thus disinhibits the LC from the inhibitory influence of the VLPO [256, 257].

##### 3.1.3.2 Paraventricular Nucleus

There is a well-defined pathway originating in the PVN and projecting to the LC [17, 54, 213, 215, 296, 331, 350, 409]. This PVN projection may form the basis of a second, indirect, pathway to the peripheral autonomic nervous system preganglionic neurones in the brainstem and spinal cord, in addition to the direct projections of the PVN to the preganglionic neurones themselves (see 2.2.2.2). CRF has been suggested as the primary neurotransmitter in the projection to the LC, since excitatory CRF immunoreactive fibres in the PVN have been found to project to, and increase the activity of, the neurones of the LC [296]. Thus, the LC appears to receive CRF inputs from both the paraventricular nucleus and the central nucleus of the amygdala (see 3.1.2).

##### 3.1.3.3 Lateral Hypothalamic/Perifornical Area

The LH/PF densely innervates the neurones of the LC [54, 193, 261] with fibres that contain the orexin peptides [74, 97, 138, 280] (see Fig. (**1**) in Part II). The orexin system originates solely in the LH/PF, with fibres projecting widely throughout the brainstem and thalamus [78]. Administration of orexin into the LC has been found to increase cell firing [74, 130], suppress REM sleep and increase wakefulness [40]. Along with excitatory projections to other brainstem wakefulness promoting nuclei, for example the TMN [21, 399], the DR [195], and cholinergic neurones of the BF [87] and LDT [45], this excitatory projection to the LC may be involved in the promotion and maintenance of wakefulness [238]. In the sleep disorder narcolepsy, the inability to maintain consistent wakefulness has been related to deficiencies in this orexinergic system [347]. In addition, the orexinergic input to the LC appears to be involved in the maintenance of muscle tone during wakefulness, since orexin microinjections into the LC have been found to facilitate muscle tone [178]. Interestingly, a case report has recently been published describing a patient with a focal lesion in the dorsomedial pontine tegmentum, involving the LC, who developed both narcolepsy and REM sleep behaviour disorder despite normal orexin levels in the cerebrospinal fluid [227]. This report, although only a single case, suggests a key role for the LC in mediating the effects of the orexinergic system on wakefulness and muscle tone.

##### 3.1.3.4 Tuberomamillary Nucleus

The wakefulness-promoting histaminergic neurones of the TMN have been found to project to the LC [149, 194] and histamine H_3_ receptors have been identified on the cell bodies of LC neurones, where they inhibit noradrenaline release [129, 281]. In contrast to other hypothalamic nuclei, the LC does not appear to project reciprocally to the TMN [95]. The neurones of the TMN are active during wakefulness and quiescent during sleep [129] and this pattern of activity is likely to result from the interaction of the TMN with the VLPO (see Figs. (**1**) and (**4**) in Part II). During sleep, when the TMN is quiescent, the inhibitory GABAergic projection from the VLPO is active [324] (see also above). In contrast, during wakefulness when the VLPO is silent (in part due to inhibition from the LC), the TMN is disinhibited and displays a high level of neurone firing [256, 257]. The inhibitory action of the TMN projection to the LC may form part of a negative feedback circuit to restrict the firing of LC neurones during wakefulness: disinhibition of the LC from VLPO neurones may otherwise lead to an ever-increasing rate of LC discharge.

#### 3.1.4 Brainstem

##### 3.1.4.1 Ventral Tegmental Area

Although the LC itself does not contain any dopaminergic cell bodies [240], dopamine-immunoreactive fibres are found within the LC [177, 219] suggesting that there is a dopaminergic projection to this area. Moreover, significant amounts of dopamine [237] and dopaminergic terminals have been found within the LC [331], and both D1-like and D2-like dopamine receptors have been identified on LC neurones [266, 348, 401]. The application of dopamine by reverse microdialysis to the vicinity of the LC inhibits sleep [68] and thus dopamine appears to have an excitatory action on the wakefulness-promoting neurones of the LC.

Neurones have been identified that project from the VTA to the LC [22, 79, 261, 266, 269, 330, 349], an area containing dopaminergic neurones involved in movement, reward, motivation and drug addiction [174, 260, 395]. Indeed, stimulation of the VTA leads to the excitation of the LC-derived noradrenergic dorsal bundle [79] whereas lesions of the VTA result in a reduction in dopamine concentration within the LC [237]. This projection, termed the “mesocoerulear” pathway, may contribute to the maintenance of arousal [305, 306, 308] (see Fig. (**1**) in Part II). Indeed, the involvement of the VTA in the promotion of wakefulness is supported by evidence indicating that the activation of the VTA produces cortical EEG desynchronisation accompanied by an increase in alertness [77].

##### 3.1.4.2 Raphe Nuclei

It has been reported that the raphe magnus in the CR may project to the LC [328]. This would form a reciprocal connection between the LC and CR for communication relating to the modulation of nociception (see 2.3.2.2). However, there are few studies relating to the outputs of the caudal raphe nuclei. In contrast, there is strong evidence that the serotonergic neurones of the DR project to the LC [54, 176, 215, 261, 276, 329, 331, 382] and this connection is likely to be related to the wakefulness-promoting roles of the two nuclei. 

##### 3.1.4.3 Pedunculopontine and Laterodorsal Tegmental Nuclei

The LC is known to receive an input from acetylcholine-releasing neurones [156, 157], and the cholinergic neurones of both the PPT and LDT have been found to project to the LC [164]. As discussed above, the PPT and LDT form two groups of neurones active during either wakefulness or REM sleep [89, 160, 171] and this cholinergic projection to the LC may thus be involved in regulating level of arousal. Perfusions of acetylcholine and cholinoceptor agonists directly into the LC increase the firing of LC neurones [86, 90] and thus increase arousal, suggesting an excitatory role for the PPT and LDT projection to the LC. 

Interestingly, the cholinergic projections of the PPT and LDT have also been implicated in the modulation of noradrenergic outputs at terminal regions. Both the noradrenergic projection to the DR [201] and the VLPO [302] are facilitated by a presynaptic cholinergic input to nicotinic receptors on the noradrenergic terminals.

##### 3.1.4.4 Periaqueductal Grey Matter

There is substantial evidence that the LC receives an input from the neurones of the PAG in the midbrain [54, 193, 215, 261, 331], particularly from the dorsolateral cell column of the PAG [49]. The precise functions of the PAG are unclear [174], but recent work has suggested a role in responses to stress, such as the “fight or flight” response, in situations of fear and anxiety [37, 179]. There are also wakefulness-active dopaminergic neurones in the ventral PAG [211] and these may be involved in the activation of LC neurones during wakefulness. In addition the ventral and ventrolateral PAG may be involved in the regulation of sleep-wakefulness state **via** inhibitory glycinergic projections to the LC [288].

An area in the subcoeruleus surrounding the LC has been identified as containing “REM-on” neurones that receive an inhibitory GABAergic projection from “REM-off” neurones in the ventrolateral PAG [212, 327]. These “REM-on” neurones in turn project to the ventrolateral PAG to inhibit the “REM-off” neurones during REM sleep episodes and may also contain GABA. This reciprocal connection has been termed a “flip-flop switch” and it has been suggested that this connection may underlie the transition into REM sleep [212, 327].

##### 3.1.4.5 Medulla 

Two groups of neurones in the rostral medulla have major inputs to the LC: the PrH [17, 93, 94, 215, 331] and the RVLM [13] (also described as the PGi) [17, 128, 215, 331, 343]. The projection from the PrH to the LC contains GABAergic neurones and is thus inhibitory to LC neurone activity [93, 94]. This GABAergic projection is likely to be involved in the inhibition of LC activity during REM sleep [381]. Indeed, electrical stimulation of GABAergic neurones in the PrH increases REM sleep duration [169] and it is likely that this occurs **via** the inhibitory projection to the wakefulness-promoting neurones of the LC. In contrast, the projection from the RVLM excites LC neurone activity **via** the release of glutamate [331] and electrical stimulation of the RVLM increases the activity within the LC [91, 92]. The projection from the RVLM to the LC is likely to be involved in the modulation of autonomic functioning, since the RVLM is known to be centrally involved in the regulation of cardiovascular function (see 2.3.2.1), and may provide an integrated input to the LC regarding this information [262, 376]. Interestingly, clonidine microinjection in the RVLM resulted in sedation in rats, suggesting that the stimulation of inhibitory α_2_-adrenoceptors on the RVLM removes the excitatory input to the LC [400]. The control of cardiovascular function and arousal are thus intricately related.

In addition to the GABAergic and glutamatergic projections to the LC from the rostral medulla, both the PrH and the PGi innervate the LC with fibres containing the endogenous opiate enkephalin [83, 155, 372]. These projections activate opiate receptors found in high concentrations in the LC to inhibit cell firing [372, 364] and the administration of endogenous opioids or opiate agonists reduces LC spontaneous firing [146, 183, 278, 371]. The LC may thus be involved in opiate-induced analgesia [83] and opiate withdrawal [155]. 

The adrenergic neurones of the ventrolateral medulla (cell groups C1-C3) also project to the LC [261], and likely to contribute to the role of the LC in cardiovascular regulation (see Section 1.2 in Part II).

#### 3.1.5 Cerebellum

There is some evidence that the nuclei of the cerebellum innervate the LC [261]. However, several reports describing the efferent projections from these nuclei do not describe terminals in the LC (for example, 190, 250, 253).

#### 3.1.6 Spinal Cord

In addition to receiving a dense innervation from the LC, the dorsal horn has been found to project to the LC in return [54, 67, 261]. It has been suggested that this pathway may communicate information relating to the detection of nociceptive and/or thermal stimuli from sensory spinal nuclei [67, 261].

### 3.2 Modulation by Autoreceptors

There are α_2_-adrenoceptors located presynaptically on LC neurones which act to inhibit the activity of these neurones [319, 358, 404] and it has been suggested that noradrenaline release from the LC is under tonic inhibitory control **via** these autoreceptors [286, 331]. Indeed, stimulation of the autoreceptors by noradrenaline application reduces LC firing rate [85, 146, 393, 394], whilst blockade of the autoreceptors increases LC activity [331] and potentiates the activity evoked by glutamate administration [147, 331]. 

The autoreceptors are endogenously activated by the release of noradrenaline from LC collaterals and thus provide a self-regulating mechanism of negative feedback [4, 85]. The autoreceptors may also be activated by noradrenaline escaping from the dendrites of LC neurones [99, 142, 285] and also by adrenaline released from an adrenergic innervation arising in the PGi of the rostral medulla [372]. Interestingly, the sleep disorder narcolepsy has been associated with an increase in autoreceptors within the LC, suggesting that an increase in LC inhibition may contribute to this disorder [109].

In addition, it has been demonstrated that μ-opiate receptors are co-localised with α_2_-adrenoceptor autoreceptors on LC neurones [5, 372] and utilise the same ion channel mediating an increase in potassium conductance [102]. Thus, these receptors also act to inhibit neurone activity within the LC (see 3.1.1.6, Part II).

## CONCLUSIONS

In conclusion, it is clear that the LC is a major noradrenergic nucleus, giving rise to fibres innervating most structures of the neuraxis in a highly specific manner. These structures control a number of physiological processes including the regulation of arousal and autonomic function and the LC is, therefore, central to the regulation of these processes. The LC is known to be a major wakefulness-promoting nucleus, with activation of the LC resulting in an increase in EEG signs of alertness. This alerting effect of LC activation results from dense excitatory projections to the majority of the cerebral cortex, wakefulness-promoting cholinergic neurones of the basal forebrain, cortically-projecting excitatory neurones of the thalamus, wakefulness-promoting serotonergic neurones of the dorsal raphe, wakefulness-promoting cholinergic neurones of the pedunculopontine tegmental nucleus and laterodorsal tegmental nucleus, and substantial inhibitory projections to sleep-promoting GABAergic neurones of the basal forebrain and ventrolateral preoptic area. It is also clear that the LC plays an important role in controlling autonomic function, where LC activation produces an increase in sympathetic activity and a concomitant decrease in parasympathetic activity. This contribution to the control of autonomic activity results both from the direct projections to the sympathetic and parasympathetic divisions of the spinal cord and from the indirect projections to various nuclei influencing the autonomic system, including the parasympathetic dorsal nucleus of the vagus and nucleus ambiguus and the sympathetic rostroventrolateral medulla, involved in cardiovascular regulation, the parasympathetic Edinger-Westphal nucleus, involved in pupil constriction, the caudal raphe, the salivatory nuclei, the paraventricular nucleus, and the amygdala. The control of arousal and autonomic function is thus inseparably linked, largely **via** the involvement of the LC. Changes in LC activity result in complex patterns of neuronal activity throughout the brain since the noradrenergic outputs from the LC can exert both excitatory effects **via** α_1_-adrenoceptors and inhibitory effects **via** α_2_-adrenoceptors. The effect of LC activation on arousal and autonomic function is therefore both interrelated and intricate, based on the compound effects of multiple projections to areas of influence.

## Abbreviations

5HT: 5-hydroxytryptamine
BF: Basal forebrain
CR: Caudal raphe
CRF: Corticotrophin-releasing factor
DMV: Dorsal motor nucleus of the vagus
DR: Dorsal raphe
EEG: Electroencephalogram
EMG: Electromyogram
EWN: Edinger-Westphal nucleus
GABA: Gamma-aminobutyric acid
GH: Growth hormone
GHRH: Growth hormone releasing hormone
IML: Intermediolateral cell column
LC: Locus coeruleus
LDT: Laterodorsal tegmental nucleus
LH/PF: Lateral hypothalamus and perifornical area
NMDA: N-methyl D-aspartate
PAG: Periaqueductal grey matter
PGi: Nucleus paragigantocellularis lateralis
PPT: Pedunculopontine tegmental nucleus
PrH: Nucleus prepositus hypoglossi
PVN: Paraventricular nucleus
REM: Rapid eye movement
RVLM: Rostroventrolateral medulla
SWS: Slow wave sleep
TMN: Tuberomamillary nucleus
VLPO: Ventrolateral preoptic area
VTA: Ventral tegmental area
